# Crystal structure of bis­{μ-2-[bis­(2-hy­droxy­eth­yl)amino]­ethano­lato}bis­(μ-3,5-di­methyl­pyrazolato)tricopper(II) dibromide sesquihydrate

**DOI:** 10.1107/S2056989020012323

**Published:** 2020-09-11

**Authors:** Oleksandr S. Vynohradov, Vadim A. Pavlenko, Dina D. Naumova, Sofiia V. Partsevska, Sergiu Shova, Safarmamad M. Safarmamadov

**Affiliations:** a Department of Chemistry, Taras Shevchenko National University of Kyiv, Volodymyrska str. 64/13, 01601 Kyiv, Ukraine; b Poni Petru Institute of Macromolecular Chemistry, Aleea Gr. Ghica, Voda 41A, 700487 Iasi, Romania; c Department of Chemistry, Tajik National University, 17, Rudaki Avenue, Dushanbe, 734025, Tajikistan

**Keywords:** copper, copper complexes, crystal structure, pyrazole, tri­ethano­lamine, X-ray crystallography, amino­alcohol ligand

## Abstract

In the title bicyclic trinuclear pyrazolate amino­alcohol complex, [Cu_3_(C_5_H_7_N_2_)_2_(C_6_H_14_NO_3_)_2_]Br_2_·1.5H_2_O, the central Cu atom lies on a center of symmetry and is involved in the formation of two five-membered rings. It has a coordination number of 4, is in a distorted tetra­hedral environment and is connected by the bridging oxygen atoms of the deprotonated OH groups of different amino­alcohol groups, and by the N atoms of deprotonated di­methyl­pyrazole ligands. The peripheral Cu atom is in a trigonal–bipyramidal coordination environment formed by the nitro­gen atom of the deprotonated bridging di­methyl­pyrazole unit, the bridging oxygen atom of the deprotonated OH group, two oxygen atoms of the protonated hy­droxy groups and the nitro­gen atom of tri­ethano­lamine. One of the C atoms and the Br^−^ anion were found to be disordered over two positions with occupancy factors of 0.808 (9):0.192 (9) and 0.922 (3):0.078 (3), respectively.

## Abstract 2

In the title bicyclic trinuclear pyrazolate amino­alcohol complex, the central Cu atom lies on a center of symmetry and has a distorted tetra­hedral coordination geometry while the peripheral Cu atom is in a trigonal–bipyramidal coordination environment.

## Chemical context   

Coordination compounds of paramagnetic transition-metal complexes with polydentate and polynuclear ligands are of great inter­est because of their versatile magnetic properties, in particular, magnetic superexchange mediated by ligand-bridging functions (Pavlishchuk *et al.*, 2010 ▸, 2011 ▸; Strotmeyer *et al.*, 2003 ▸; Gumienna-Kontecka *et al.*, 2007 ▸) or spin-crossover behavior (Suleimanov *et al.*, 2015 ▸; Gural’skiy *et al.*, 2012 ▸). Amino alcohols can be used for the synthesis of similar complexes since they are versatile and effective polydentate ligands in coordination chemistry (Vynohradov *et al.*, 2020 ▸). It is well known that polynuclear complexes of 3*d* metals with amino alcohols (acting both as neutral and acidic ligands) can indicate non-trivial magnetic properties and biological activity. Mono-, di-, and trinuclear complexes of copper(II) with tri­ethano­lamine are widely studied because of their inter­esting magnetic properties (Escovar *et al.*, 2005 ▸). The magnetic properties of copper(II) complexes with tri­ethano­lamine range from ferromagnetic to anti­ferromagnetic, with minor changes in the structure of the complex affecting the nature of the exchange inter­actions that control the ultimate magnetization (Boulsourani *et al.*, 2011 ▸). In addition, copper(II) complexes with tri­ethano­lamine can bind to DNA (Sama *et al.*, 2019 ▸) and show catecholase activity (Sama *et al.*, 2017 ▸). Amino alcohol complexes of copper(II) and zinc show catalytic activity in the reactions of conversion of alkanes or cyclo­alkanes to carb­oxy­lic acids, which can help to increase the yield of products (Ansari *et al.*, 2016 ▸ *)*. Tri­ethano­lamine is a polyfunctional *O*,*N*-ligand that can bind metal ions in its neutral or deprotonated form leading to an alcoholate. Finally, atoms of the same or different metals can be linked by bridging oxygen atoms to form mono- and heterometallic polynuclear complexes (Dias *et al.*, 2015 ▸; Kirillov *et al.*, 2007 ▸). As part of our continuing inter­est in multifunctional transition-metal complexes with polydentate and polynuclear ligands, we report herein the synthesis and crystal structure of a new trinuclear copper(II) mixed-ligand complex with tri­ethano­lamine and 3,5-di­methyl­pyrazole.
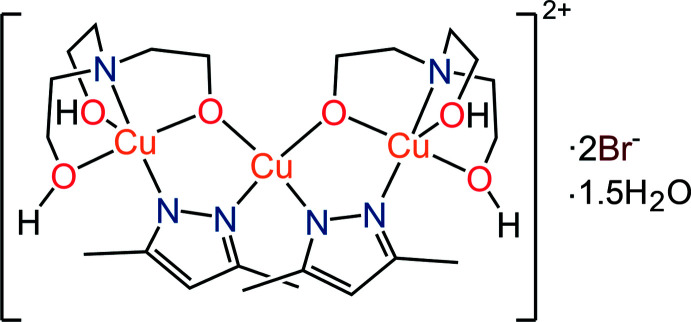


## Structural commentary   

**Figure 1:**
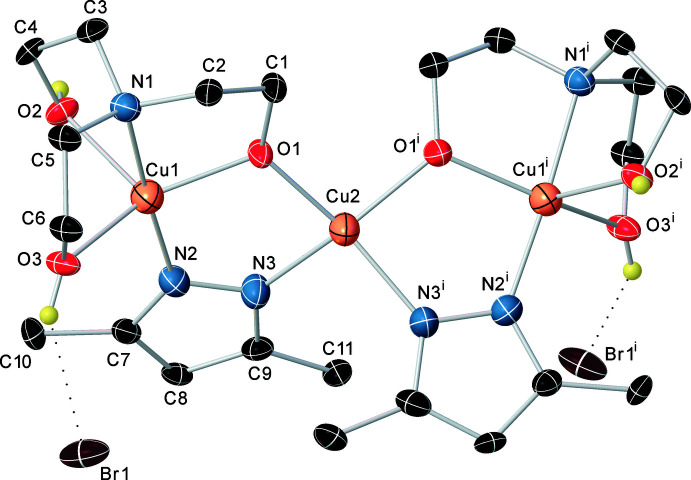
The mol­ecular structure of the title compound with displacement ellipsoids drawn at the 50% probability level [Symmetry code: (i) 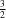 − *x*, *y*, 1 − *z*].

The crystal structure of the title compound (Fig. 1 ▸) comprises trinuclear Cu_3_(dmpz-H)_2_(H_2_TEA)_2_^2+^ cationic units linked *via* two bridging bromine anions. The central Cu2 atom lies on a center of symmetry and is involved in the formation of two five-membered rings. Each ring is formed by two copper atoms connected by the bridging oxygen atom of the monodeprotonated tri­ethano­lamine and the bridging deprotonated di­methyl­pyrazole. The five-membered bimetallic rings are not planar. The nitro­gen atoms of the di­methyl­pyrazole bridging ligand are practically in the same plane as the metal atoms, while the bridging oxygen atom is out of the plane by 0.450 (3) Å. The copper(II) atoms have different coordination environments. The peripheral Cu1 atom is in a trigonal–bipyramidal coordination environment formed by two N2 nitro­gen atoms of the deprotonated bridging di­methyl­pyrazole ligands, the bridging oxygen atom of the deprotonated OH group, two oxygen atoms of the protonated hy­droxy groups and the tri­ethano­lamine nitro­gen atom. The central Cu2 atom (coordination number 4) is in a distorted (flattened) tetra­hedral environment and is surrounded by the bridging oxygen atoms of the deprotonated OH groups of different amino alcohol mol­ecules, and by N3 and N3^i^ symmetry code: (i) 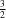 − *x*, *y*, 1 − *z*] atoms of different deprotonated mol­ecules of dimethyl­pyrazole. The inter­atomic distances between the N3, O1 and N3^i^, O1^i^ atoms are 2.726 (4) Å. The distances between the atoms O1, O1^i^ and N3, N3^i^ are similar at 2.915 (4) and 2.970 (5) Å, respectively. The inter­metallic separations are Cu1⋯Cu2 = 3.2829 (5) and Cu1⋯Cu1^i^ = 6.4784 (10) Å.

The tri­ethano­lamine ligand is coordinated in a tetra­dentate manner by all donor atoms. As a result of such a coordination of tri­ethano­lamine from both sides of the complex mol­ecule, three similar five-membered cyclic Cu–O–C–C–N fragments are formed. Bridging oxygen atoms arise from the coordination of the amino alcohol to a metal atom with the deprotonation of only one OH group. The coordinated tri­ethano­lamine is monodeprotonated, and the other two hy­droxy groups are protonated and bonded by hydrogen bonds to the adjacent mol­ecules *via* bridging bromine anions. The distances between Cu1 and the oxygen atoms of the deprotonated [Cu1—O1 = 1.930 (2) Å] and protonated [Cu1—O2 = 2.308 (2), Cu1—O3 = 2.060 (3) Å] OH groups are different.

## Supra­molecular features   

**Figure 2:**
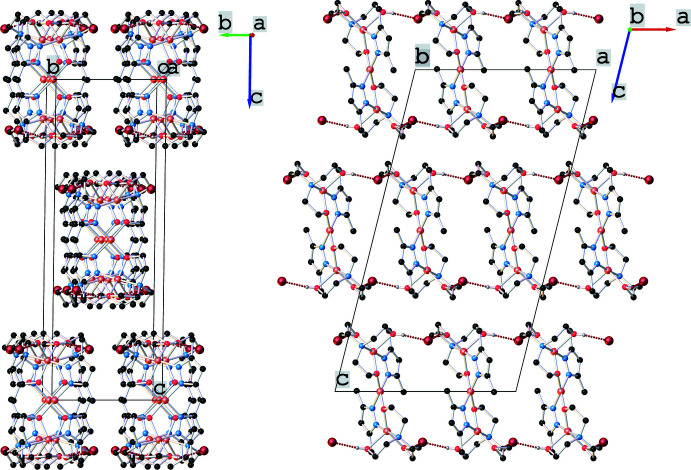
Crystal packing of the title compound viewed along the *a*- (left) and *b*-axis (right) directions.

In the crystal, the trinuclear cationic complexes inter­act *via* O—H⋯Br hydrogen bonding (Table 1 ▸), forming one-dimensional supra­molecular networks. The distances between copper atoms within the supra­molecular chain are Cu1⋯Cu1(−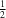 + *x*, 1 − *y*, *z*) = 7.3123 (4) Å, Cu2⋯Cu2(−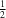 + *x*, 1 − *y*, *z*) = 7.2470 (4) Å, Cu1(−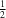 + *x*, 1 − *y*, *z*)⋯Cu1(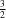 − *x*, *y*, 1 − *z*) = 8.9185 (12) Å, and Cu1⋯Cu1(1 − *x*, 1 − *y*, 1 − *z*) = 10.5517 (10) Å. The crystal structure is built up from the parallel packing of discrete pillars along the *a* axis (Fig. 2 ▸). The co-crystallized water mol­ecules, which are fractionally disordered over several positions, fill the voids formed in the crystal and do not contribute significantly to extending the hydrogen-bonded network.

## Database survey   

A search of the Cambridge Structural Database (CSD version 5.40, update of August 2019; Groom *et al.*, 2016 ▸) for the Cu(HO-CH_2_CH_2_)(O-CH_2_CH_2_)_2_N moiety revealed 171 hits. Most similar to the title compound are the trinuclear complexes with coordinated tri­ethano­lamine and other ligands [WISQOH, WISQUN (Sun *et al.*, 2018 ▸); AWEQEZ, AWEQID, AWEQOJ, AWEQUP (Boulsourani *et al.*, 2011 ▸); DEGSOX (Ferguson *et al.*, 1985 ▸); FISJIB (Tudor *et al.*, 2005 ▸); KUDYUF (Dias *et al.*, 2015 ▸); MEDHUZ, MEDHUZ01, MEDJAH, MEDJEL, MEDJIP (Escovar *et al.*, 2005 ▸); OYALEH02 (Ansari *et al.*, 2016 ▸); ZACTIJ01, ZAGYIS (Ozarowski *et al.*, 2015 ▸)].

## Synthesis and crystallization   

**Figure 3:**
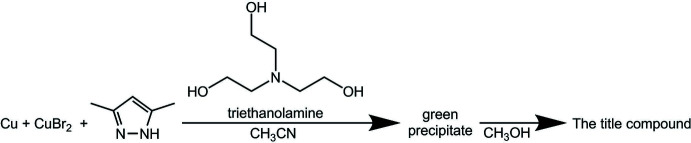
Reaction scheme to obtain the title compound.

Cu_3_(dmpz-H)_2_(H_2_TEA)_2_Br_2_ (dmpz-H = deprotonated 3,5-dimethyl-1*H*-pyrazole and H_2_TEA = monodeprotonated tri­ethano­lamine) was synthesized at room temperature by the addition of a copper powder (2.34 mmol, 0.15 g) and copper(II) bromide (2.34 mmol, 0.525 g) mixture to an aceto­nitrile solution of 3,5-dimethyl-1*H*-pyrazole (4.68 mmol, 0.45 g). Tri­ethano­lamine (2.34 mmol, 0.31 ml) was added immediately. The reaction mixture was stirred without heating for one h with free air access until dissolution of the copper powder, and a green precipitate of the product was obtained. The precipitate was filtered off, dissolved in methanol, and filtered off from the undissolved copper residues. Green crystals suitable for X-ray analysis were obtained by slow evaporation of the solvent. The yield was 50%. The obtained dark-green crystals were studied by elemental analysis (calculated C 31.56%, H 5.05% and N 10.04%, found C 30.83%, H 5.73%, N 10.38%). The reaction scheme is shown in Fig. 3 ▸.

## Refinement   

Crystal data, data collection and structure refinement details are summarized in Table 2 ▸. Hydrogen atoms were included in geometrically calculated positions (O—H = 0.83–0.88 Å, C—H = 0.96–0.97 Å) with *U*_iso_ = 1.2*U*_eq_C) or *U*_iso_ = 1.5*U*_eq_(O,C-meth­yl). Atom C6 and the Br^−^ anion were found to be disordered over two resolvable positions with occupancy factors of 0.808 (9):0.192 (9) and 0.922 (3):0.078 (3), respectively. Their positional parameters were refined using available tools (see the CIF in the supporting information).

## Supplementary Material

Crystal structure: contains datablock(s) I. DOI: 10.1107/S2056989020012323/dj2014sup1.cif

Structure factors: contains datablock(s) I. DOI: 10.1107/S2056989020012323/dj2014Isup5.hkl

here is the mol file of structural formula of the title compound. DOI: 10.1107/S2056989020012323/dj2014sup3.mol

here is the IR spectrum of the title compound. DOI: 10.1107/S2056989020012323/dj2014sup4.txt

CCDC reference: 2030043

Additional supporting information:  crystallographic information; 3D view; checkCIF report

## Figures and Tables

**Table 1:** Hydrogen-bond geometry (Å, °)

*D*—H⋯*A*	*D*—H	H⋯*A*	*D*⋯*A*	*D*—H⋯*A*
O2—H2⋯Br1^i^	0.83	2.50	3.288 (3)	158
O3—H3*B*⋯Br1*X*	0.85	2.37	3.207 (8)	168

Symmetry code: (i) 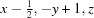.

**Table 2:** Experimental details

Crystal data	
Chemical formula	[Cu_3_(C_5_H_7_N_2_)_2_(C_6_H_14_NO_3_)_2_]Br_2_·1.5H_2_O
*M*_r_	864.08
Crystal system, space group	Monoclinic, *I*2/*a*
Temperature (K)	293
*a*, *b*, *c* (Å)	14.4930 (7), 8.8855 (3), 26.6017 (11)
β (°)	103.998 (5)
*V* (Å^3^)	3324.0 (2)
*Z*	4
Radiation type	Mo *K*α
μ (mm^−1^)	4.35
Crystal size (mm)	0.25 × 0.15 × 0.15

Data collection	
Diffractometer	Rigaku Oxford Diffraction Xcalibur, Eos
Absorption correction	Multi-scan (*CrysAlis PRO*; Rigaku OD, 2019 ▸)
*T*_min_, *T*_max_	0.514, 1.000
No. of measured, independent and observed [*I* > 2σ(*I*)] reflections	10477, 3946, 3296
*R*_int_	0.024
(sin θ/λ)_max_ (Å^−1^)	0.689

Refinement	
*R*[*F* ^2^ > 2σ(*F* ^2^)], *wR*(*F* ^2^), *S*	0.039, 0.105, 1.09
No. of reflections	3946
No. of parameters	199
No. of restraints	11
H-atom treatment	H-atom parameters constrained
Δρ_max_, Δρ_min_ (e Å^−3^)	0.61, −0.52

Computer programs: *CrysAlis PRO* (Rigaku OD, 2019 ▸), *SHELXT* (Sheldrick, 2015*a* ▸), *SHELXL2018/3* (Sheldrick, 2015*b* ▸) and *OLEX2* (Dolomanov *et al.*, 2009 ▸).

## supplementary crystallographic information

### Crystal data

**Table:**

[Cu_3_(C_5_H_7_N_2_)_2_(C_6_H_14_NO_3_)_2_]Br_2_·1.5H_2_O	*F*(000) = 1744
*M**_r_* = 864.08	*D*_x_ = 1.727 Mg m^−^^3^
Monoclinic, *I*2/*a*	Mo *K*α radiation, λ = 0.71073 Å
*a* = 14.4930 (7) Å	Cell parameters from 4599 reflections
*b* = 8.8855 (3) Å	θ = 1.6–27.9°
*c* = 26.6017 (11) Å	µ = 4.35 mm^−^^1^
β = 103.998 (5)°	*T* = 293 K
*V* = 3324.0 (2) Å^3^	Block, dark green
*Z* = 4	0.25 × 0.15 × 0.15 mm

### Data collection

**Table:**

Rigaku Oxford Diffraction Xcalibur, Eos diffractometer	3946 independent reflections
Radiation source: fine-focus sealed X-ray tube, Enhance (Mo) X-ray Source	3296 reflections with *I* > 2σ(*I*)
Graphite monochromator	*R*_int_ = 0.024
Detector resolution: 8.0797 pixels mm^-1^	θ_max_ = 29.3°, θ_min_ = 2.4°
ω scans	*h* = −16→18
Absorption correction: multi-scan (CrysAlisPro; Rigaku OD, 2019)	*k* = −9→11
*T*_min_ = 0.514, *T*_max_ = 1.000	*l* = −35→36
10477 measured reflections	

### Refinement

**Table:**

Refinement on *F*^2^	Primary atom site location: dual
Least-squares matrix: full	Hydrogen site location: mixed
*R*[*F*^2^ > 2σ(*F*^2^)] = 0.039	H-atom parameters constrained
*wR*(*F*^2^) = 0.105	*w* = 1/[σ^2^(*F*_o_^2^) + (0.0485*P*)^2^ + 4.2091*P*] where *P* = (*F*_o_^2^ + 2*F*_c_^2^)/3
*S* = 1.09	(Δ/σ)_max_ = 0.001
3946 reflections	Δρ_max_ = 0.61 e Å^−^^3^
199 parameters	Δρ_min_ = −0.52 e Å^−^^3^
11 restraints	

### Special details

**Table:**

Geometry. All esds (except the esd in the dihedral angle between two l.s. planes) are estimated using the full covariance matrix. The cell esds are taken into account individually in the estimation of esds in distances, angles and torsion angles; correlations between esds in cell parameters are only used when they are defined by crystal symmetry. An approximate (isotropic) treatment of cell esds is used for estimating esds involving l.s. planes.

### Fractional atomic coordinates and isotropic or equivalent isotropic displacement parameters (Å^2^)

**Table:**

	*x*	*y*	*z*	*U*_iso_*/*U*_eq_	Occ. (<1)
Cu1	0.84276 (3)	0.55507 (4)	0.62368 (2)	0.03014 (12)	
Cu2	0.750000	0.49500 (6)	0.500000	0.03372 (15)	
O1	0.79457 (19)	0.6382 (3)	0.55533 (8)	0.0434 (6)	
O2	0.75819 (19)	0.6565 (3)	0.67875 (10)	0.0492 (6)	
H2	0.701401	0.678302	0.675390	0.074*	
O3	0.9595 (2)	0.4666 (3)	0.67533 (11)	0.0571 (7)	
H3A	0.991686	0.384510	0.679523	0.086*	0.808 (9)
H3B	0.986816	0.387400	0.668063	0.086*	0.192 (9)
N1	0.91464 (19)	0.7483 (3)	0.64151 (10)	0.0336 (6)	
N2	0.77343 (19)	0.3725 (3)	0.60337 (9)	0.0316 (5)	
N3	0.72982 (19)	0.3495 (3)	0.55213 (9)	0.0323 (6)	
C1	0.8348 (3)	0.7810 (4)	0.54973 (13)	0.0494 (10)	
H1A	0.790451	0.859727	0.553258	0.059*	
H1B	0.846751	0.789021	0.515481	0.059*	
C2	0.9267 (3)	0.8016 (4)	0.59024 (13)	0.0460 (8)	
H2A	0.976989	0.744829	0.580605	0.055*	
H2B	0.944604	0.907048	0.592508	0.055*	
C3	0.8581 (3)	0.8578 (4)	0.66391 (15)	0.0480 (9)	
H3C	0.809372	0.901971	0.636341	0.058*	
H3D	0.899332	0.938101	0.680996	0.058*	
C4	0.8128 (3)	0.7830 (5)	0.70191 (16)	0.0561 (10)	
H4A	0.861466	0.750289	0.731725	0.067*	
H4B	0.771818	0.854094	0.713755	0.067*	
C5	1.0076 (3)	0.7209 (5)	0.67819 (16)	0.0555 (10)	
H5BC	1.056188	0.726257	0.658782	0.067*	0.192 (9)
H5BD	1.019202	0.803170	0.702872	0.067*	0.192 (9)
H5AA	1.054166	0.790854	0.671037	0.067*	0.808 (9)
H5AB	1.001831	0.738938	0.713262	0.067*	0.808 (9)
C7	0.7491 (2)	0.2588 (4)	0.63086 (12)	0.0363 (7)	
C8	0.6888 (3)	0.1623 (4)	0.59740 (14)	0.0406 (8)	
H8	0.661023	0.074739	0.606027	0.049*	
C9	0.6785 (2)	0.2229 (4)	0.54863 (13)	0.0365 (7)	
C10	0.7851 (3)	0.2510 (5)	0.68850 (13)	0.0552 (10)	
H10A	0.848281	0.210080	0.696948	0.083*	
H10B	0.744002	0.187678	0.702649	0.083*	
H10C	0.786095	0.350270	0.702877	0.083*	
C11	0.6195 (3)	0.1634 (5)	0.49835 (16)	0.0575 (11)	
H11A	0.559290	0.214164	0.489894	0.086*	
H11B	0.609514	0.057368	0.501520	0.086*	
H11C	0.652047	0.180536	0.471421	0.086*	
C6	1.0402 (3)	0.5685 (5)	0.6742 (3)	0.0616 (16)	0.808 (9)
H6A	1.093912	0.546357	0.702993	0.074*	0.808 (9)
H6B	1.060015	0.555500	0.642187	0.074*	0.808 (9)
C6B	1.0213 (14)	0.5817 (9)	0.7073 (4)	0.0616 (16)	0.192 (9)
H6BA	1.004131	0.594819	0.740059	0.074*	0.192 (9)
H6BB	1.087428	0.550866	0.714297	0.074*	0.192 (9)
Br1	1.05467 (4)	0.16214 (7)	0.65764 (5)	0.0663 (3)	0.922 (3)
O1W	1.0873 (10)	0.3397 (16)	0.5606 (5)	0.070 (3)*	0.25
H1WA	1.038994	0.394250	0.558614	0.106*	0.25
H1WB	1.082024	0.278840	0.584344	0.106*	0.25
Br1X	1.0615 (5)	0.1928 (9)	0.6317 (5)	0.0663 (3)	0.078 (3)
O2W	1.0549 (11)	0.4390 (18)	0.5497 (6)	0.082 (4)*	0.25
H2WA	0.996778	0.470066	0.536178	0.123*	0.25
H2WB	1.043988	0.373266	0.571568	0.123*	0.25
O3W	0.9438 (11)	0.5317 (17)	0.5068 (6)	0.081 (4)*	0.25
H3WA	0.954628	0.626818	0.515273	0.122*	0.25
H3WB	1.002938	0.507648	0.511103	0.122*	0.25

### Atomic displacement parameters (Å^2^)

**Table:**

	*U*^11^	*U*^22^	*U*^33^	*U*^12^	*U*^13^	*U*^23^
Cu1	0.0351 (2)	0.0281 (2)	0.02443 (19)	−0.00146 (15)	0.00192 (15)	0.00083 (14)
Cu2	0.0532 (4)	0.0248 (3)	0.0211 (2)	0.000	0.0048 (2)	0.000
O1	0.0705 (17)	0.0284 (12)	0.0251 (10)	−0.0126 (12)	−0.0006 (11)	0.0021 (9)
O2	0.0461 (15)	0.0502 (16)	0.0552 (15)	−0.0009 (12)	0.0198 (12)	−0.0090 (12)
O3	0.0548 (16)	0.0369 (14)	0.0650 (17)	0.0088 (12)	−0.0140 (14)	−0.0003 (13)
N1	0.0327 (14)	0.0316 (14)	0.0333 (13)	−0.0008 (11)	0.0017 (11)	−0.0030 (11)
N2	0.0372 (14)	0.0304 (13)	0.0255 (12)	−0.0024 (11)	0.0043 (10)	0.0038 (10)
N3	0.0405 (15)	0.0288 (13)	0.0249 (12)	−0.0033 (11)	0.0027 (11)	0.0005 (10)
C1	0.079 (3)	0.0348 (19)	0.0291 (16)	−0.0169 (19)	0.0029 (17)	0.0028 (14)
C2	0.056 (2)	0.0382 (19)	0.0451 (19)	−0.0124 (17)	0.0148 (17)	0.0001 (16)
C3	0.053 (2)	0.041 (2)	0.050 (2)	−0.0022 (17)	0.0127 (18)	−0.0158 (17)
C4	0.058 (2)	0.062 (3)	0.051 (2)	0.001 (2)	0.0181 (19)	−0.019 (2)
C5	0.038 (2)	0.056 (2)	0.061 (2)	−0.0050 (18)	−0.0104 (18)	0.007 (2)
C7	0.0411 (18)	0.0321 (17)	0.0385 (17)	0.0058 (14)	0.0150 (14)	0.0088 (14)
C8	0.044 (2)	0.0293 (17)	0.052 (2)	−0.0003 (14)	0.0186 (16)	0.0068 (15)
C9	0.0369 (17)	0.0279 (16)	0.0434 (18)	−0.0016 (14)	0.0069 (14)	−0.0009 (14)
C10	0.079 (3)	0.051 (2)	0.0374 (19)	0.001 (2)	0.0176 (19)	0.0151 (17)
C11	0.058 (3)	0.047 (2)	0.060 (2)	−0.0145 (19)	0.001 (2)	−0.0107 (19)
C6	0.042 (3)	0.054 (3)	0.078 (4)	0.004 (2)	−0.007 (3)	−0.014 (3)
C6B	0.042 (3)	0.054 (3)	0.078 (4)	0.004 (2)	−0.007 (3)	−0.014 (3)
Br1	0.0543 (3)	0.0459 (3)	0.0959 (6)	−0.0023 (2)	0.0127 (3)	−0.0174 (3)
Br1X	0.0543 (3)	0.0459 (3)	0.0959 (6)	−0.0023 (2)	0.0127 (3)	−0.0174 (3)

### Geometric parameters (Å, º)

**Table:**

Cu1—O1	1.930 (2)	C4—H4A	0.9700
Cu1—O2	2.308 (2)	C4—H4B	0.9700
Cu1—O3	2.060 (3)	C5—H5BC	0.9700
Cu1—N1	2.005 (3)	C5—H5BD	0.9700
Cu1—N2	1.916 (3)	C5—H5AA	0.9700
Cu2—O1	1.935 (2)	C5—H5AB	0.9700
Cu2—O1^i^	1.935 (2)	C5—C6	1.447 (6)
Cu2—N3	1.969 (2)	C5—C6B	1.447 (6)
Cu2—N3^i^	1.969 (2)	C7—C8	1.384 (5)
O1—C1	1.420 (4)	C7—C10	1.497 (5)
O2—H2	0.8291	C8—H8	0.9300
O2—C4	1.426 (5)	C8—C9	1.379 (5)
O3—H3A	0.8581	C9—C11	1.499 (5)
O3—H3B	0.8520	C10—H10A	0.9600
O3—C6	1.485 (5)	C10—H10B	0.9600
O3—C6B	1.485 (6)	C10—H10C	0.9600
N1—C2	1.493 (4)	C11—H11A	0.9600
N1—C3	1.486 (4)	C11—H11B	0.9600
N1—C5	1.480 (4)	C11—H11C	0.9600
N2—N3	1.372 (3)	C6—H6A	0.9700
N2—C7	1.343 (4)	C6—H6B	0.9700
N3—C9	1.340 (4)	C6B—H6BA	0.9700
C1—H1A	0.9700	C6B—H6BB	0.9700
C1—H1B	0.9700	O1W—H1WA	0.8426
C1—C2	1.508 (5)	O1W—H1WB	0.8490
C2—H2A	0.9700	O2W—H2WA	0.8768
C2—H2B	0.9700	O2W—H2WB	0.8660
C3—H3C	0.9700	O3W—H3WA	0.8786
C3—H3D	0.9700	O3W—H3WB	0.8643
C3—C4	1.489 (6)	O3W—H3WB^ii^	1.06 (3)
			
O1—Cu1—O2	108.77 (11)	C4—C3—H3C	109.5
O1—Cu1—O3	145.70 (12)	C4—C3—H3D	109.5
O1—Cu1—N1	86.79 (10)	O2—C4—C3	110.4 (3)
O3—Cu1—O2	101.67 (11)	O2—C4—H4A	109.6
N1—Cu1—O2	80.85 (10)	O2—C4—H4B	109.6
N1—Cu1—O3	82.72 (11)	C3—C4—H4A	109.6
N2—Cu1—O1	90.73 (10)	C3—C4—H4B	109.6
N2—Cu1—O2	100.76 (10)	H4A—C4—H4B	108.1
N2—Cu1—O3	98.91 (11)	N1—C5—H5BC	107.7
N2—Cu1—N1	177.38 (10)	N1—C5—H5BD	107.7
O1—Cu2—O1^i^	97.76 (13)	N1—C5—H5AA	109.3
O1—Cu2—N3	88.57 (10)	N1—C5—H5AB	109.3
O1—Cu2—N3^i^	152.70 (11)	H5BC—C5—H5BD	107.1
O1^i^—Cu2—N3^i^	88.57 (10)	H5AA—C5—H5AB	108.0
O1^i^—Cu2—N3	152.70 (11)	C6—C5—N1	111.6 (4)
N3—Cu2—N3^i^	97.91 (15)	C6—C5—H5AA	109.3
Cu1—O1—Cu2	116.31 (11)	C6—C5—H5AB	109.3
C1—O1—Cu1	112.13 (19)	C6B—C5—N1	118.6 (6)
C1—O1—Cu2	125.31 (19)	C6B—C5—H5BC	107.7
Cu1—O2—H2	133.5	C6B—C5—H5BD	107.7
C4—O2—Cu1	104.9 (2)	N2—C7—C8	108.9 (3)
C4—O2—H2	107.1	N2—C7—C10	121.4 (3)
Cu1—O3—H3A	138.1	C8—C7—C10	129.7 (3)
Cu1—O3—H3B	121.0	C7—C8—H8	127.1
C6—O3—Cu1	106.1 (3)	C9—C8—C7	105.7 (3)
C6—O3—H3A	96.6	C9—C8—H8	127.1
C6B—O3—Cu1	113.8 (5)	N3—C9—C8	109.3 (3)
C6B—O3—H3B	116.7	N3—C9—C11	123.0 (3)
C2—N1—Cu1	102.98 (19)	C8—C9—C11	127.7 (3)
C3—N1—Cu1	110.5 (2)	C7—C10—H10A	109.5
C3—N1—C2	110.9 (3)	C7—C10—H10B	109.5
C5—N1—Cu1	110.7 (2)	C7—C10—H10C	109.5
C5—N1—C2	111.4 (3)	H10A—C10—H10B	109.5
C5—N1—C3	110.2 (3)	H10A—C10—H10C	109.5
N3—N2—Cu1	119.36 (19)	H10B—C10—H10C	109.5
C7—N2—Cu1	132.2 (2)	C9—C11—H11A	109.5
C7—N2—N3	108.1 (3)	C9—C11—H11B	109.5
N2—N3—Cu2	119.40 (19)	C9—C11—H11C	109.5
C9—N3—Cu2	132.6 (2)	H11A—C11—H11B	109.5
C9—N3—N2	108.0 (2)	H11A—C11—H11C	109.5
O1—C1—H1A	109.6	H11B—C11—H11C	109.5
O1—C1—H1B	109.6	O3—C6—H6A	110.3
O1—C1—C2	110.3 (3)	O3—C6—H6B	110.3
H1A—C1—H1B	108.1	C5—C6—O3	107.2 (4)
C2—C1—H1A	109.6	C5—C6—H6A	110.3
C2—C1—H1B	109.6	C5—C6—H6B	110.3
N1—C2—C1	109.6 (3)	H6A—C6—H6B	108.5
N1—C2—H2A	109.7	O3—C6B—H6BA	110.3
N1—C2—H2B	109.7	O3—C6B—H6BB	110.3
C1—C2—H2A	109.7	C5—C6B—O3	107.3 (4)
C1—C2—H2B	109.7	C5—C6B—H6BA	110.3
H2A—C2—H2B	108.2	C5—C6B—H6BB	110.3
N1—C3—H3C	109.5	H6BA—C6B—H6BB	108.5
N1—C3—H3D	109.5	H1WA—O1W—H1WB	101.0
N1—C3—C4	110.9 (3)	H2WA—O2W—H2WB	100.0
H3C—C3—H3D	108.0	H3WA—O3W—H3WB	95.4
			
Cu1—O1—C1—C2	20.5 (4)	N2—N3—C9—C8	−0.4 (4)
Cu1—O2—C4—C3	35.7 (4)	N2—N3—C9—C11	178.9 (3)
Cu1—O3—C6—C5	47.4 (5)	N2—C7—C8—C9	0.3 (4)
Cu1—O3—C6B—C5	−25.7 (17)	N3—N2—C7—C8	−0.5 (4)
Cu1—N1—C2—C1	43.4 (3)	N3—N2—C7—C10	−179.8 (3)
Cu1—N1—C3—C4	43.4 (4)	C2—N1—C3—C4	156.9 (3)
Cu1—N1—C5—C6	25.0 (5)	C2—N1—C5—C6	−88.9 (4)
Cu1—N1—C5—C6B	−18.7 (10)	C2—N1—C5—C6B	−132.6 (10)
Cu1—N2—N3—Cu2	6.0 (3)	C3—N1—C2—C1	−74.8 (4)
Cu1—N2—N3—C9	−173.7 (2)	C3—N1—C5—C6	147.6 (4)
Cu1—N2—C7—C8	172.7 (2)	C3—N1—C5—C6B	103.8 (10)
Cu1—N2—C7—C10	−6.6 (5)	C5—N1—C2—C1	162.0 (3)
Cu2—O1—C1—C2	−130.5 (3)	C5—N1—C3—C4	−79.3 (4)
Cu2—N3—C9—C8	180.0 (2)	C7—N2—N3—Cu2	−179.7 (2)
Cu2—N3—C9—C11	−0.7 (5)	C7—N2—N3—C9	0.6 (3)
O1—C1—C2—N1	−43.7 (4)	C7—C8—C9—N3	0.0 (4)
N1—C3—C4—O2	−54.2 (4)	C7—C8—C9—C11	−179.2 (4)
N1—C5—C6—O3	−48.3 (6)	C10—C7—C8—C9	179.5 (4)
N1—C5—C6B—O3	29.0 (18)		

Symmetry codes: (i) −*x*+3/2, *y*, −*z*+1; (ii) −*x*+2, −*y*+1, −*z*+1.

### Hydrogen-bond geometry (Å, º)

**Table:**

*D*—H···*A*	*D*—H	H···*A*	*D*···*A*	*D*—H···*A*
O2—H2···Br1^iii^	0.83	2.50	3.288 (3)	158
O3—H3*B*···Br1*X*	0.85	2.37	3.207 (8)	168

Symmetry code: (iii) *x*−1/2, −*y*+1, *z*.
